# Calpastatin phosphorylation regulates radiation-induced calpain activity in glioblastoma

**DOI:** 10.18632/oncotarget.24523

**Published:** 2018-02-19

**Authors:** Emily A. Bassett, Kamalakannan Palanichamy, Mitchell Pearson, Joseph P. McElroy, Saikh Jaharul Haque, Erica Hlavin Bell, Arnab Chakravarti

**Affiliations:** ^1^ Department of Radiation Oncology, The Ohio State University Wexner Medical Center, Columbus, OH, USA; ^2^ Center for Biostatistics, Department of Biomedical Informatics, The Ohio State University, Columbus, OH, USA

**Keywords:** glioblastoma, phospho-proteomic profiling, radiation response, calpastatin, casein kinase 2

## Abstract

Glioblastoma (GBM) is an aggressive, malignant brain tumor that inevitably develops resistance to conventional chemotherapy and radiation treatments. In order to identify signaling pathways involved in the development of radiation resistance, we performed mass spectrometry-based phospho-proteomic profiling of GBM cell lines and normal human astrocytes before and after radiation treatment. We found radiation induced phosphorylation of a number of proteins including calpastatin, specifically in GBM stem cells (GSCs). Herein, we focused on calpastatin, an endogenous inhibitor of calpain proteases. Radiation-induced phosphorylation of calpastatin at Ser-633 within the inhibitory domain was validated with a phospho-specific antibody. In order to test the functional significance of phosphorylated calpastatin, we utilized site-directed mutagenesis to generate phospho-inactive (Ser633Ala) and phospho-mimetic (Ser633Glu) mutant calpastatin. GBM cell lines stably expressing the mutant calpastatin showed that phosphorylation was necessary for radiation-induced calpain activation. We also showed that casein kinase 2, a pro-survival kinase overexpressed in many cancer types, phosphorylated calpastatin at Ser-633. Our results indicate that calpastatin phosphorylation promotes radiation resistance in GBM cells by increasing the activity of calpain proteases, which are known to promote survival and invasion in cancer.

## INTRODUCTION

Glioblastoma (GBM) is the most prevalent and aggressive primary malignant brain tumor in adults, accounting for 60–70% of malignant gliomas. GBM is a grade IV malignant glioma with 17,000 new cases diagnosed each year in the United States [1]. Patients diagnosed with GBM have a very poor prognosis and quality of life, with a median survival time of 12–15 months despite receiving the standard care that includes maximally safe resection followed by radiotherapy plus concomitant and adjuvant temozolomide chemotherapy [2]. GBMs are highly heterogeneous, invasive, and resistant to both chemo- and radiotherapy [3–4]. Many studies have shown that knockdown or pharmacological inhibition of specific proteins within canonical signaling pathways, such as DNA damage checkpoint activation and repair [5–7] and the Akt pathway [8], can increase the radiosensitivity of GBM cells. Despite extensive studies on molecules regulating GBM radioresistance, the cellular mechanisms responsible for GBM treatment resistance remain largely unknown. This lack of progress is in part due to the fact that most of the data on these signaling pathways has been obtained pre-treatment, not accounting for how treatment-induced changes in signaling can impact therapeutic resistance. Certain signaling pathways appear to be associated with the phenomenon of radiation resistance; however, there is relatively sparse knowledge of the mechanisms by which radiation activates these pathways in GBMs, or how activation of these pathways leads to radiation resistance.

In this study, we used label-free, mass spectrometry (MS)-based phospho-proteomic profiling to identify proteins involved in early response to radiation treatment in GBM. We focused on phosphorylation because it is the most common mechanism of regulating protein function and signal transduction, providing fast and highly regulated responses to stimuli. Phospho-proteomic profiling can reveal alterations in protein phosphorylation that have a direct effect on protein function and would not be detected by genomic or transcriptomic profiling. We profiled an established GBM cell line as well as primary GSCs before and after radiation treatment in order to identify treatment-induced changes in phosphorylation. GSCs represent a distinct population of tumor cells that possess the ability to proliferate, self-renew, differentiate, and initiate and maintain tumors. A growing amount of data suggests that GSCs are highly resistant to conventional treatments compared to the bulk of differentiated tumor cells [5, 9]. We present data which identified a novel role of calpastatin phosphorylation in the radiation response of GSCs.

Calpastatin is an endogenous inhibitor of the calpain family of cysteine proteases. Calpains regulate a diverse range of signaling pathways by limited proteolysis of target proteins, including cell cycle (cyclin D1 and cyclin E), cell survival (nuclear factor-κB), migration (focal adhesion kinase and talin), and apoptosis (BCL-2 family and caspases) [10] (Supplementary Figure 1). Out of the 15 known calpains, calpain 1 and calpain 2 are the most studied and are ubiquitously expressed. Calpains 1 and 2 bind to the regulatory subunit calpain S1 to form heterodimers that were originally named μ-calpain and m-calpain, respectively [11]. Inhibition of calpain proteases is the only known function of calpastatin. Calpastatin contains an N-terminal L-domain that is subject to alternative splicing, as well as four repetitive inhibitory domains that each binds to and inhibits one calpain heterodimer. Each calpastatin inhibitory domain consists of 3 subdomains, designated A, B, and C, with conserved sequences [12]. Phosphorylation of calpastatin leads to aggregation and inhibition of the protein [13–14]. Reversible inhibition of calpastatin via phosphorylation has been proposed as a mechanism to regulate calpain activation.

Altered regulation of the calpastatin-calpain proteolytic system is associated with several pathological disorders, including cancer [15]. The conventional calpains (calpain 1, calpain 2, and calpain S1) show consistent overexpression or increased activity across many cancer types including gliomas [16–22], breast cancer [23], colorectal adenocarcinoma [18], renal carcinoma [17], and rhabdomyosarcoma [24]. Calpastatin levels, however, vary across cancer types as the protein is reported to have high expression in endometrial cancer [25] and low expression in rhabdomyosarcoma [24]. Despite a clear involvement of the calpastatin-calpain system in cancer, its contribution to treatment resistance has not yet been explored. Here, we present data identifying a novel role for calpastatin phosphorylation in the regulation of calpain activity following radiation treatment. This work provides new insights into signaling pathways that contribute to radiation treatment resistance in glioblastoma.

## RESULTS

### Phospho-proteomic profiling of normal human astrocytes and GBM cell lines

**Figure 1:**
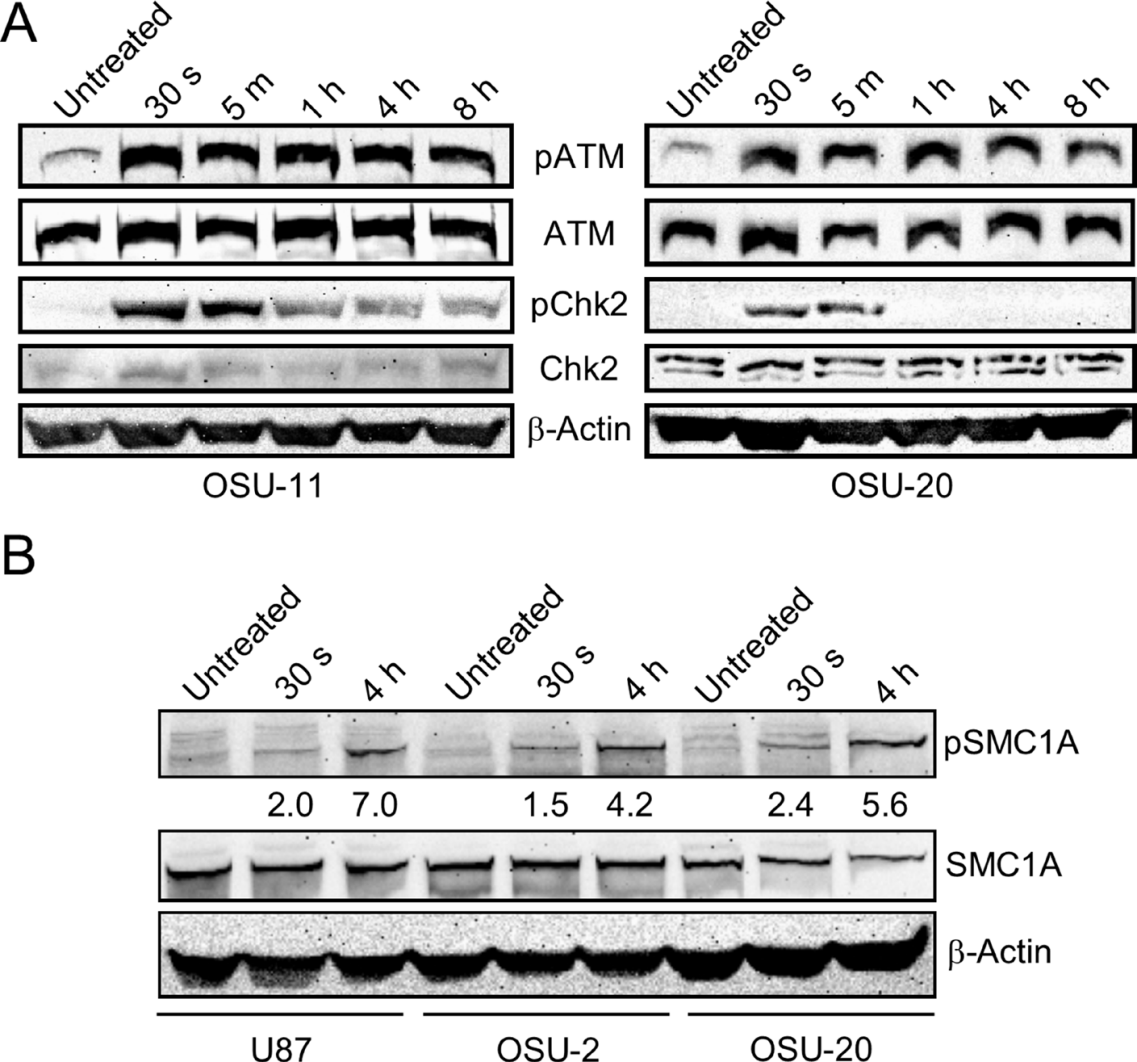
Optimization of phospho-proteomic profiling of GBM cell lines (**A**) Two GSC lines, OSU-11 and OSU-20, were treated with 10 Gy radiation and frozen at various time-points. Western blotting with phospho-specific antibodies for ATM (Ser 1981) and Chk2 (Thr 68) reveals the changes in phosphorylation over time. Antibodies for the non-phosphorylated proteins were used to monitor total protein levels over time. (**B**) Western blot of phospho-SMC1A (Ser 957) and total SMC1A before and after radiation treatment in three GBM cell lines. The fold changes in phosphorylation calculated from mass spectrometry for SMC1A-pS957 are shown below the corresponding lanes in the blot.

In order to identify radiation-induced changes in protein phosphorylation, we first performed phospho-proteomic profiling of normal human astrocytes (NHAs) before and after radiation treatment. Post-radiation time-points of 30 seconds and 4 hours were chosen for profiling based on western blots of proteins in the DNA damage response pathway showing the temporal dynamics of phosphorylation (Figure 1A). Cells were collected in triplicate before treatment, and at 30 s and 4 h time-points after 10 Gy radiation treatment. Following protein isolation and phospho-peptide enrichment, global phospho-proteomic analysis was performed by MS (Supplementary Figure 2). We quantified 1,925 phospho-peptides mapping to 894 unique proteins that are common to both the 30 s and 4 h time-point datasets for NHAs. Relative quantitation and statistical analysis were performed to determine fold changes in phosphorylation at both post-radiation time-points (30 s/untreated and 4 h/untreated). The quantified MS dataset was then filtered for peptides with a fold change of at least 1.5 (up- or down-regulation) and a *p* value less than 0.05.

We then performed phospho-proteomic profiling of 4 GBM cell lines with the same radiation treatment and time-points as used above for NHA. One differentiated cell line (U87 monolayer) and 3 primary GSC lines (OSU-2, OSU-11, and OSU-20) were profiled in a single batch, with biological triplicates of each condition. Mass spectrometry results showed a phospho-peptide enrichment efficiency of 95%. We quantified 6,398 phospho-peptides mapping to 2,416 unique proteins that were common across all 36 samples. This includes 5,574 serine, 913 threonine, and 156 tyrosine residues that were found to be phosphorylated. To validate our phospho-proteomics data for GBM cell lines, we performed a Western analysis of SMC1A, a protein involved in DNA repair and known to be phosphorylated at serine-957 by ATM following radiation treatment [26]. An increase in phospho-SMC1A was evident at 4 h post-radiation in all cell lines tested, which was consistent with fold changes in phosphorylation calculated from the MS data (Figure 1B). Additional proteins known to be phosphorylated at specific residues in response to radiation treatment were identified in our phospho-proteomics data, including mediator of DNA damage checkpoint protein 1 (MDC1) [27], tumor protein p53 binding protein 1 (TP53BP1) [28], and tripartite motif containing 28 (TRIM28) [29] (Supplementary Tables 1 and 2).

**Table 1:** Post-radiation fold changes in phosphorylation at calpastatin serines 351 and 633

Calpastatin Phospho-peptide	Phosphosite	Post-RT Time-point	OSU-2	OSU-11	OSU-20	U87	NHA
KPADDQDPIDAL[S]GDLDSCPSTTETSQNTAK	S633	30 s	5.0^*^	4.2	3.4^*^	−1.6	N/A
KPADDQDPIDAL[S]GDLDSCPSTTETSQNTAK	S633	4 h	6.3^*^	3.5	3.2^*^	−1.7	N/A
SESELIDEL[S]EDFDR	S351	30 s	2.2^*^	2.4	2.7^*^	1.6	N/A
SESELIDEL[S]EDFDR	S351	4 h	3.4^*^	3.4^*^	3.5^*^	2.0	N/A

Fold changes were calculated for each time-point (30 s/Untreated and 4 h/Untreated) from triplicate intensity values from mass spectrometry data. Asterisk indicates a *p*-value of < 0.05. N/A indicates that no calpastatin peptides were identified in NHA. The phosphorylated residue in the phospho-peptide is indicated in brackets.

As described above, statistical analysis was performed to identify significant changes in phosphorylation at 30 s and 4 h post-radiation. The number of phospho-peptides with significant fold-changes in each cell line ranged from 105 to 349 at the 30 s time-point, and from 216 to 251 at the 4 h time-point. Because GSCs are typically more resistant to radiation treatment than bulk tumor cells, we compared phospho-peptides that are altered post-radiation in the 3 GSCs but not U87. Conversely, we also identified phospho-peptides altered in U87 but not GSCs because these changes might uncover pathways that were improperly silenced in GSCs following treatment. Ingenuity pathway analysis of the phospho-peptides differentially regulated in GSCs versus U87 following radiation treatment was performed.

The top pathway altered specifically in GSCs following radiation treatment was “regulation by calpain protease”. Within this pathway, we found that calpastatin protein is consistently phosphorylated across the 3 GSCs. Two different calpastatin peptides showed significant increases in phosphorylation at one or both time-points across all GSCs (Table 1). Fold changes in calpastatin phosphorylation in U87 were minimal and not statistically significant for either phospho-peptide. In order to validate radiation-induced phosphorylation of calpastatin in GSCs, one additional primary GSC line (OSU-53) was profiled by MS. OSU-53 was found to have statistically significant increases in phosphorylation at the 4 h time-point in the same two calpastatin phospho-peptides (Supplementary Table 3). Calpastatin phospho-peptides were not identified in the NHAs by MS. Western blotting revealed that total calpastatin protein levels were significantly reduced in NHA compared to GBM cell lines (Supplementary Figure 4C).

### Validation of radiation-induced phosphorylation of calpastatin

**Figure 2:**
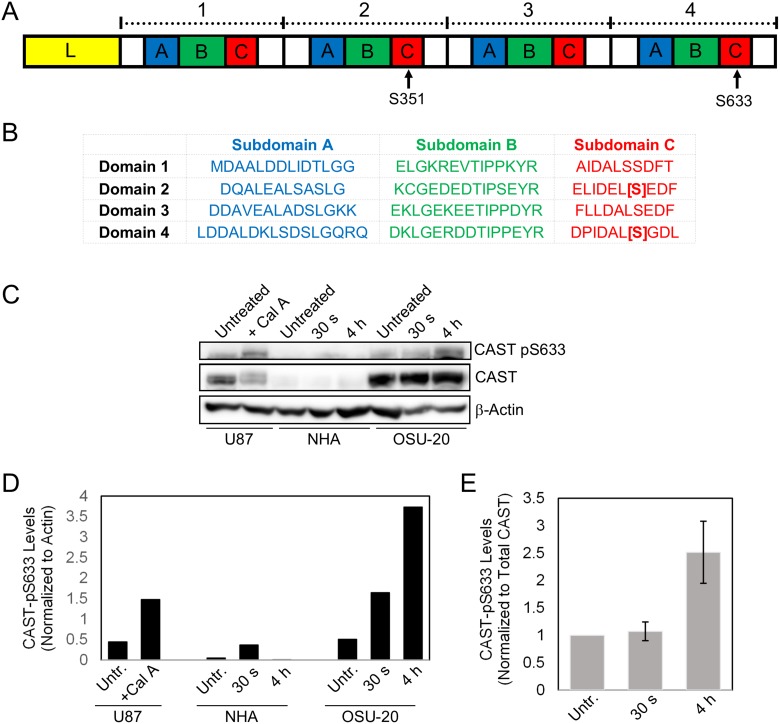
Validation of radiation-induced phosphorylation of calpastatin in GSCs (**A**) Calpastatin protein consists of an N-terminal linker domain (L), and four repetitive inhibitory domains. Each inhibitory domain contains subdomains A, B, and C. Serines 351 and 633 are located in inhibitory domains 2 and 4, respectively. (**B**) The sequences of the four inhibitory domains of calpastatin show that serines 351 and 633 are located at homologous sites in subdomain C. (**C**) Western blot of NHA and OSU-20 cell lines at 30 seconds and 4 hours following 10 Gy radiation treatment, using a custom phospho-specific antibody against calpastatin-pS633. U87 cells were treated with Calyculin A, a phosphatase inhibitor, as a positive control for detection of calpastatin-pS633. (**D**) Quantitation of phosho-calpastatin from the western blot in (C), normalized to the loading control beta-actin. (**E**) Quantitation of phosho-calpastatin in OSU-20 from the western blot in (C), normalized to total calpastatin levels. CAST = calpastatin. Untr. = Untreated.

Calpastatin was chosen for further validation and mechanistic studies because it functions upstream of multiple signaling pathways implicated in cancer, is altered in all four GSC lines post-radiation treatment, and has not been previously reported to be involved in radiation resistance mechanisms. Further analysis of the two significantly different calpastatin phospho-peptides revealed that the phosphorylated residues were serines 351 and 633 (with 98.8% and 94.4% localization confidence, respectively). Interestingly, these two residues are located at homologous sites within subdomain C of inhibitory domains 2 and 4, respectively (Figure 2A, 2B) [12].

In order to validate the radiation-induced calpastatin phosphorylation observed by MS, phospho-specific antibodies were generated for serines 351 and 633. The phospho-antibodies were tested on U87 cell extracts treated with or without calyculin A, a serine/threonine phosphatase inhibitor that enriches for phosphorylated calpastatin by stabilizing the modification. Western analysis with the calpastatin-pS633 antibody revealed a distinct band upon calyculin A treatment at 120 KDa, which corresponded to the molecular weight of calpastatin (Supplementary Figure 3B). However, the calpastatin-pS351 antibody failed to detect increased calpastatin phosphorylation and was not used for further experiments (Supplementary Figure 3A). Next, we used the calpastatin-pS633 antibody to test post-radiation levels of calpastatin phosphorylation in OSU-20 and NHA cell lines. OSU-20 cells showed a marked increase in phospho-calpastatin at both time-points following 10 Gy radiation treatment (Figure 2C–2D and Supplementary Figure 4A–4B). Levels of phospho-calpastatin were quantified and normalized to total calpastatin levels, to ensure that the observed increase in calpastatin phosphorylation was not due to an increase in total protein (Figure 2E). Calpastatin phosphorylation at serine 633 was not detected in NHA cells, even when twice as much total protein was loaded compared to OSU-20 (Supplementary Figure 4D). Total calpastatin levels were found to be much lower in NHAs compared to OSU-20 (Supplementary Figure 4A, 4C), which was consistent with TCGA data showing lower calpastatin gene expression in normal brain tissue compared to GBM tumors (Supplementary Figure 5).

### Calpastatin phosphorylation is required for post-radiation calpain activation

**Figure 3:**
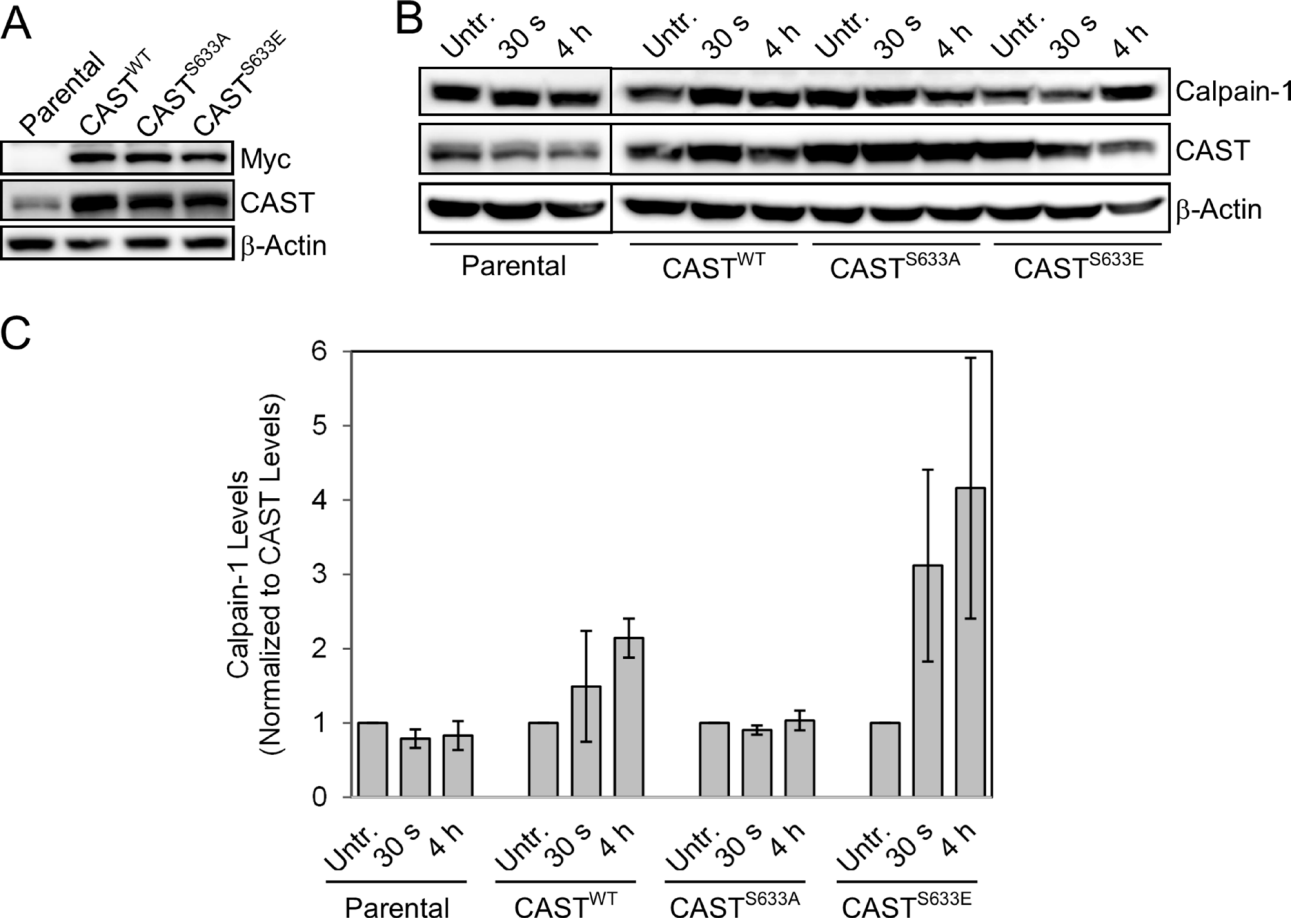
Functional analysis of calpastatin phosphorylation using site-directed mutagenesis (**A**) Stable cell lines expressing calpastatin^WT^, calpastatin^S655A^ (non-phosphorylatable mutant), or calpastatin^S655E^ (phospho-mimetic mutant) were generated in LN18 GBM cells. (**B**) Western blot of calpain-1 levels in each of the calpastatin stable cell lines and parental LN18 cells 30 seconds and 4 hours after a 10 Gy radiation treatment. (**C**) Quantitation of calpain-1 levels from two independent western blots (shown in (B) and Supplementary Figure 6A) normalized to total calpastatin. CAST = calpastatin. Untr. = Untreated.

The functional significance of calpastatin phosphorylation at serines 351 or 633 has not yet been reported. Previous studies show that phosphorylation of calpastatin at other residues leads to reversible aggregation near the nucleus [13–14]. In addition, the only known function of calpastatin is to inhibit calpain proteases. Based on this, we hypothesized that increased calpastatin phosphorylation at serines 351 and 633 following radiation treatment in GSCs leads to inhibition of calpastatin activity, thereby increasing the calpain activity. In order to test this hypothesis, we utilized site-directed mutagenesis to generate a non-phosphorylatable mutant (calpastatin^S633A^) and a phospho-mimetic mutant (calpastatin^S633E^). Serine 633 was chosen for functional studies because phosphorylation of this residue was validated by Western analysis. Stable cell lines overexpressing myc-tagged calpastatin^WT^, calpastatin^S633A^, or calpastatin^S633E^ were generated in two established GBM cell lines, LN18 and U87 (Figure 3A, Supplementary Figure 7A).

Calpain is known to be activated in cancer cell lines as early as 15 minutes following treatment with ionizing radiation [30]. We reasoned that the effects of calpastatin phosphorylation on calpain activity may not be evident until after radiation treatment. Therefore, we treated the stable cell lines and parental LN18 cells with 10 Gy radiation, and collected cells at 30 second and 4 hour time-points. Levels of calpain 1 were measured by quantitation of Western blots from duplicate experiments, normalizing to calpastatin to rule out the possibility that changes in calpain levels post-radiation were due to unequal levels of its inhibitor (Figure 3B, 3C and Supplementary Figure 6A). The cell line expressing the phospho-mimetic calpastatin mutant showed increased levels of calpain following radiation treatment. This was consistent with our hypothesis that phosphorylation of calpastatin reduces its inhibitory activity, leading to activation of calpain. Conversely, the non-phosphorylatable calpastatin mutant cell line showed no changes in calpain levels post-radiation, suggesting that non-phosphorylated calpastatin inhibited calpain and prevented its activation. We also observed higher post-radiation calpain levels with calpastatin^S633E^ overexpression compared to calpastatin^S633A^ in U87 cells (Supplementary Figure 7). When levels of the regulatory subunit calpain-S1 were compared in the calpastatin^S633A^ and calpastatin^S633E^ stable cell lines, no difference was observed (Supplementary Figure 6A, 6B). This demonstrates that calpastatin phosphorylation specifically regulates calpain-1.

### Casein kinase 2 phosphorylates calpastatin

**Figure 4:**
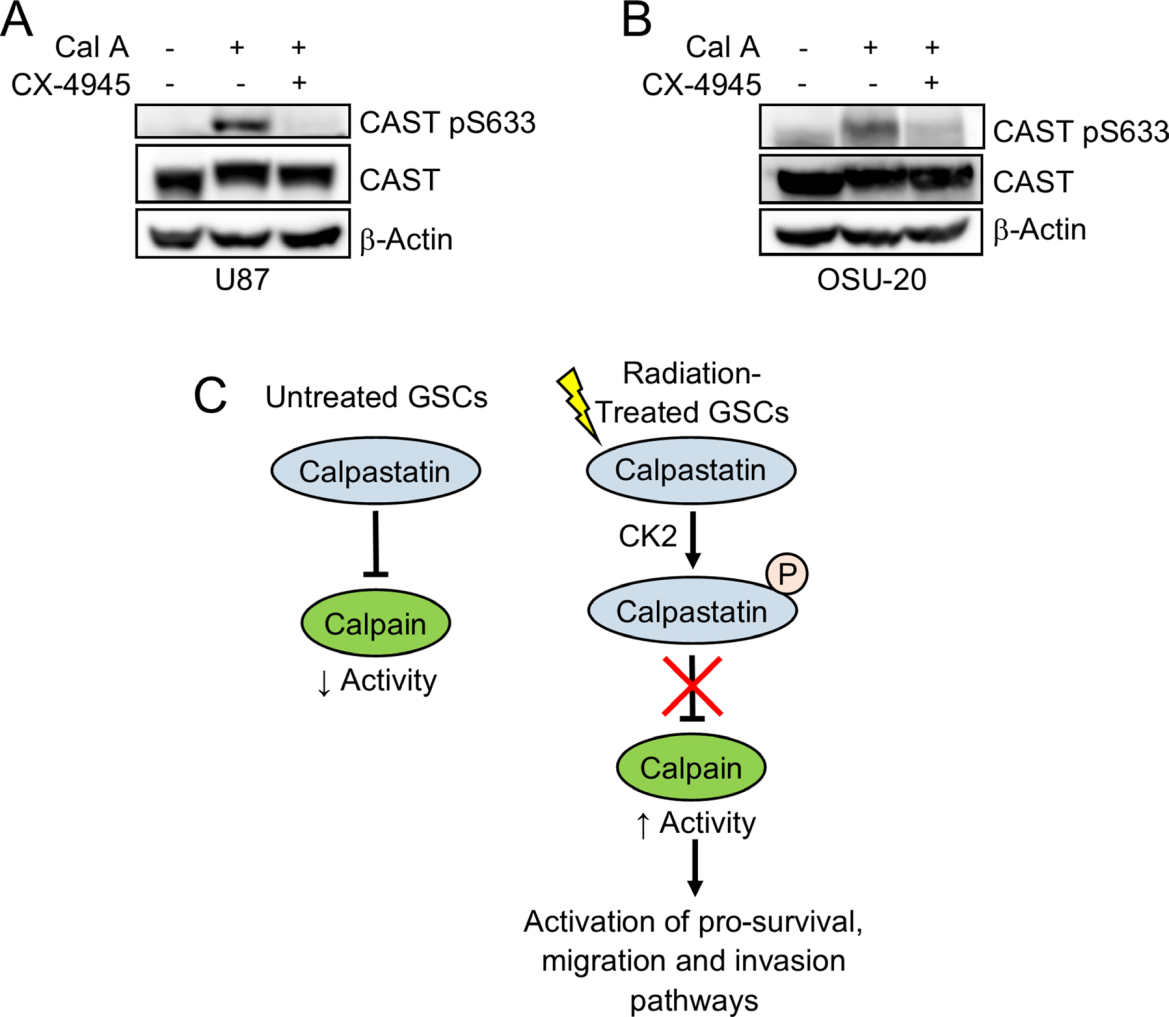
Casein kinase 2 phosphorylates calpastatin at serine 633 Western blot of U87 cells (**A**) and OSU-20 GSCs (**B**) treated with or without the phosphatase inhibitor calyculin A, and with or without the casein kinase 2 inhibitor CX-4945. (**C**) A model for the role of calpastatin in the radiation response of GSCs. Non-phosphorylated calpastatin inhibits calpain in untreated GSCs. Upon radiation treatment, casein kinase 2 phosphorylates calpastatin at serine 633, which inhibits calpastatin activity and leads to increased calpain activity. CAST = calpastatin.

In order to identify the kinase that phosphorylates calpastatin specifically at serines 633 and 351, we utilized two sequence-based kinase prediction tools (KinasePhos [31] and NetPhos 3.1 [32]). Casein kinase 2 (CK2) was predicted by both tools to phosphorylate calpastatin serines 633 and 351, and has previously been reported to play a role in GSC maintenance [33]. To test the hypothesis that CK2 phosphorylates calpastatin at Ser-633, we treated cells with the CK2 inhibitor CX-4945 and calyculin A. Treatment with calyculin A alone enriched for phospho-calpastatin at Ser-633 through stabilization. However, 24 h pre-treatment with CX-4945 inhibited the stabilization of phospho-calpastatin by calyculin A, suggesting that CK2 phosphorylates calpastatin at Ser-633 (Figure 4A, 4B). We propose a model in which calpastatin phosphorylation contributes to radiation resistance in GSCs (Figure 4C). Upon radiation treatment, CK2 phosphorylates calpastatin at specific serines within subdomain C of the inhibitory domain, which blocks its inhibitory activity. Disruption of the calpastatin-calpain complex leads to activation of calpain, a protease that has been shown to upregulate pro-survival, migration, and invasion pathways.

## DISCUSSION

Our phospho-proteomic profiling data of normal human astrocytes and GBM cell lines demonstrated that changes in protein phosphorylation could be detected immediately following radiation treatment, and some of these changes were specific to GSCs. Because protein phosphorylation is one of the primary mechanisms regulating signaling transduction, we hypothesized that radiation-induced changes in phosphorylation occurring specifically in GSCs contribute to treatment resistance mechanisms. Supporting this idea, our pathway analysis identified PI3K/AKT signaling as one of the top pathways differentially regulated between GSCs and U87 following radiation treatment. We previously reported that activation of the PI3K/AKT pathway is associated with radiation resistance and adverse outcome in glioma patients [34–35]. In addition to validating pathways previously reported to be involved in radiation resistance, our results identified novel proteins potentially involved in resistance mechanisms. In this study, we focused on calpastatin which was found to be phosphorylated specifically in GSCs following radiation. Future analysis of the phosphorylation events that occur in U87 or NHA but not in GSCs may also provide insight into the mechanisms of radiation resistance, as these events may indicate signaling pathways that are improperly altered in the radiation-resistant GSCs.

Our results suggest that radiation-induced calpastatin phosphorylation was specific to GSCs. We found a significant increase in calpastatin phosphorylation following radiation treatment in all four GSCs profiled, including one GSC profiled in an independent experiment, but not in U87. The absence of calpastatin phosphorylation in U87 cells could alternatively be explained by underlying genetic alterations in U87 that differ from the 4 GSCs profiled. No calpastatin peptides were identified by MS in NHAs, which suggests that calpastatin is not phosphorylated post-radiation in these cells. However, Western analysis showed that calpastatin total protein abundance was very low in NHAs relative to OSU-20 (Figure 2C, Supplementary Figure 4C), so it is also possible that calpastatin was phosphorylated in NHAs but at very low levels that were under the detection limit of the mass spectrometer.

Our MS results indicated that homologous serines in inhibitory domains 2 and 4 (serine 351 and 633, respectively) of calpastatin were phosphorylated in GSCs following radiation treatment. The calpastatin inhibitory domains have conserved sequences and can each bind and inhibit one calpain molecule. Although phospho-proteomic profiling did not reveal phosphorylation of the homologous serines in inhibitory domains 1 and 3 (serines 214 and 494, respectively), we propose that all four sites are phosphorylated and have identical functions. Phospho-proteomic profiling by mass spectrometry is a sampling technique that cannot comprehensively identify every phosphorylation event in the cell, which may explain the absence of phospho-peptides spanning serines 214 and 494. Also, our analysis here of radiation-induced changes in phosphorylation is limited to two early time-points, and it is possible that additional calpastatin residues are phosphorylated later than 4 hours post-radiation.

In order to determine if calpastatin phosphorylation at serine 633 has an effect on calpain activity, we mutated the residue to an alanine to mimic constitutively non-phosphorylated calpastatin. Consistent with our hypothesis, blockage of calpastatin phosphorylation in the LN18 calpastatin^S633A^ mutant cell line prevented an increase in calpain protein levels (Figure 3). Calpastatin phosphorylation appears to specifically regulate the calpain-1 large subunit, as we did not observe a similar trend with calpain-S1 (Supplementary Figure 6A, 6B). Our results indicate that calpastatin phosphorylation is necessary but not sufficient for calpain activation, because mutation of calpastatin serine 633 to alanine only affected calpain protein levels in the context of radiation treatment (Figure 3 and Supplementary Figure 6). Consistent with this idea, calpain activation has been shown to require an increase in cytosolic calcium levels that triggers conformational changes [36]. Thus, calpain activation is likely a multi-step process and disruption of the calpastatin/calpain complex by phosphorylation of calpastatin is one of these steps. We propose a model in which radiation treatment induces calpastatin phosphorylation by casein kinase 2 in GSCs, which subsequently leads to the release of calpain. Calpain is then available to be activated through other mechanisms likely involving calcium. In differentiated GBM cells, in which calpastatin was not found to be phosphorylated following radiation treatment, we propose that calpastatin remains active and inhibits calpain.

Increased calpain activity is known to be associated with malignant transformation and tumor progression. Calpain activity has been shown to be increased by the oncoproteins v-Myc, v-Src, k-Ras and v-Fos during cell transformation [11]. Knockdown of calpastatin, which would lead to increased calpain activity, has been shown to promote transformation of c-Myc-deficient cells [37]. However, this is the first study to report that increased calpain activity via alteration of its endogenous inhibitor is involved in radiation resistance. There are likely multiple different mechanisms of radiation resistance that differ for each tumor and cancer type. Some pathways, such as PI3K/AKT, seem to be consistently associated with radiation resistance across many cancer types. We found that calpastatin was consistently phosphorylated in all 4 GSCs profiled, suggesting that alteration of the calpastatin/calpain proteolytic system is a common resistance mechanism in GBMs. However, future studies are needed to determine if calpastatin contributes to radiation resistance in other cancers as well.

Pre-treatment of GBM cells with a casein kinase 2 inhibitor prevented calyculin A-induced accumulation of phospho-calpastatin at Ser-633 (Figure 4A, 4B), suggesting that CK2 is the kinase that phosphorylates calpastatin post-radiation. Endogenous levels of phosphorylated calpastatin are too low to be detected by western blot in the cell lines tested, so calyculin A was used as a tool to enrich for phospho-calpastatin via stabilization of the modification. Casein kinase 2 is elevated in GBM tumors and 33% of GBMs were found to have gene dosage gains in *CSNK2A1*, the catalytic subunit of CK2 [38–39]. CK2 has been implicated in GSC growth and maintenance [33], which may explain why calpastatin phosphorylation was observed in GSCs post-radiation, but not U87. CK2 also has a role in the early radiation response and regulates proteins involved in DNA double-strand break repair [40–41], which is consistent with our model that CK2 phosphorylates calpastatin soon after radiation treatment.

Clinical trials are in progress to test the efficacy of the casein kinase inhibitor CX-4945 in multiple cancer types such as multiple myeloma and cholangiocarcinoma (clinicaltrials.gov). A preclinical study showed that CX-4945 treatment suppresses prosurvival pathways and decreases migration of GBM, demonstrating the potential utility of targeting the CK2 pathway in GBM patients [39]. Studies have also shown promise for calpain inhibitors in certain cancers, such as calpeptin that was shown to reduce tumor volume in pancreatic cancer xenografts [42]. Most calpain inhibitors developed to date target the active site, which is conserved among cysteine proteases. For this reason, calpain inhibitors lack specificity and often target multiple proteases. It has been suggested that alternative methods for inhibiting calpain that do not involve the enzyme's active site may be more therapeutically relevant [43]. Our results showing that blockage of calpastatin phosphorylation prevents calpain activation suggest that modulation of calpastatin activity is an attractive alternative to targeting calpain itself, especially considering that calpain is the only known target of calpastatin. We observed a change in calpain levels post-radiation when a single serine in one of the four calpastatin inhibitory domains was mutated. Considering that each calpastatin inhibitory domain inhibits one calpain molecule, the effect of blocking calpastatin phosphorylation is expected to be much more pronounced if all four of the homologous serines across all inhibitory domains are mutated. In conclusion, we have identified calpastatin as a potential therapeutic target to prevent radiation resistance in GBM. Further studies will be required to determine if targeting calpastatin is more effective than targeting calpain.

## MATERIALS AND METHODS

### Cell culture

U87 cell line was purchased from ATCC and maintained in DMEM (Life Technologies), supplemented with 10% FBS (Sigma-Aldrich), and 1% antibiotic-antimycotic (Life Technologies). The 4 primary glioblastoma stem cell lines (OSU-2, −11, −20, and −53) were isolated from glioblastoma patient tissues and authenticated by a neuropathologist at The Ohio State University. GSCs were maintained in DMEM:F12 (Life Technologies) supplemented with B27 supplement (Gibco), recombinant human FGF (Gibco), recombinant human EGF (Gibco), 10% FBS and 1% antibiotic-antimycotic (Life Technologies). Normal human astrocytes were obtained from Lonza and maintained in Clonetics AGM Astrocyte Growth Medium (Lonza) supplemented with 10% FBS and 1% antibiotic-antimycotic (Life Technologies). All cells were cultured at 37°C under a gas phase of 95% air and 5% CO_2_. For calyculin A treatment, cells were treated with 100 nM calyculin A (Santa Cruz) for 30 minutes, and then harvested for western blot. For combination calyculin A and CX-4945 treatment, cells were treated with 10 μM CX-4945 (Selleckchem) for 24 hours, followed by 100 nM calyculin A for 30 minutes.

### Radiation treatments

U87 cells were plated to be ~80% confluent on the day of radiation. GSCs were plated as single cell suspensions and incubated for 5 days to form neurospheres, and were plated at various cell numbers in order to have similar cell densities on the day of radiation. Cells were irradiated with a 10 gray dose using a RS-2000 biological irradiator (RadSource). At 30 seconds or 4 hours following treatment, cells were centrifuged at 4°C and washed twice with ice cold PBS. Cell pellets (containing 3–4 × 10^6^ cells) were frozen in liquid nitrogen.

### Phospho-proteomic profiling

Cell pellets were thawed on ice and resuspended in 2% SDS containing1% v/v protease inhibitor cocktail (Sigma-Aldrich), 1% v/v phosphatase inhibitor cocktail 2 (Sigma-Aldrich), and 1% v/v phosphatase inhibitor cocktail 3 (Sigma-Aldrich). Following a 30 minute incubation on ice, cells were sonicated on ice with a probe sonicator (Branson) at 10% power for 30 seconds. Lysates were vortexed until completely solubilized. Filter-Aided Sample Preparation (FASP) clean-up was then performed according to Wisniewski [44] to remove detergent from samples. Briefly, protein lysates were exchanged into 8 M urea using a 10K MWCO centrifugal filter (Millipore). Lysates were incubated with 10 mM DTT (Sigma) in filter for 1 hour at 37°C, followed by 25 mM iodoacetamine (Sigma) in filter for 30 minutes at room temperature. Eight hundred micrograms of each sample were digested overnight with sequencing grade trypsin (Promega) at a 1:30 ratio in 50 mM Tris pH 8. Following digestion, the pH of samples was reduced to < 3.0 using 20% TFA. C18 clean-up was performed with a silica C18 spin column (The Nest Group). Samples were then run in a speed-vac evaporator until dry. Phospho-peptide enrichment was performed on 400 μg of each sample with the Pierce TiO_2_ phospho-peptide enrichment kit (ThermoFisher Scientific). LC-MS/MS data collection was then performed for each sample using a UPLC system (NanoAcquity, Waters) interfaced to an Orbitrap ProVelos Elite MS system (ThermoFisher Scientific). All peptide precursor ions across all chromatographic analyses were clustered using the peak alignment algorithm of Rosetta Elucidator software. Automated differential quantification of phospho-peptides was accomplished using downstream quantitative analysis modules of the Rosetta Elucidator. Peptide and protein identifications were integrated from the protein database search engine output (MASCOT, Matrix Science Inc.) with these quantifications.

### Statistical and pathway analysis

Within each batch, data was quantile normalized to remove overall sample effects. For each peptide within each cell line, geometric fold changes were calculated for both time-points (30 s versus untreated, and 4 h versus untreated). Paired t tests (paired by replicate, *N* = 3) were performed for each fold change. Data was filtered for peptides with a fold change > 1.5 in either direction (corresponding to a ratio of < 0.66 or > 1.5), and a *p*-value < 0.05. Significant peptides were imported into Ingenuity Pathway Analysis (Qiagen). A core analysis was performed to identify the top canonical pathways with differential phosphorylation.

### Site-directed mutagenesis

Calpastatin human cDNA ORF clone (transcript variant 2) was purchased from Origene.

QuikChange II XL Site-Directed Mutagenesis Kit (Agilent Technologies) was used to introduce point mutations into calpastatin. Mutagenesis primers for calpastatin S633A mutant are CCC ATT GAT GCT CTC GCA GGA GAT CTG GAC AGC (forward) and GCT GTC CAG ATC TCC TGC GAG AGC ATC AAT GGG (reverse). Mutagenesis primers for calpastatin S633E mutant are CCC ATT GAT GCT CTC GAA GGA GAT CTG GAC AGC TG (forward) and CAG CTG TCC AGA TCT CCT TCG AGA GCA TCA ATG GG (reverse).

### Generation of stable cell lines

DNA plasmids for calpastatin^WT^, calpastatin^S633A^, calpastatin^S633E^, and pCMV6-Entry empty vector (Origene) were transfected into U87 and LN18 cells using Lipofectamine 3000 (Invitrogen). Selection was performed using 0.5 mg/mL Geneticin (Gibco) until no cells remained on the empty vector plate. Calpastatin protein expression was assessed by Western analysis following completion of selection. Stable cell lines were maintained with 0.5 mg/mL Geneticin.

### Western analysis

Protein was extracted from cells using RIPA buffer (Sigma-Aldrich) with 1% v/v protease inhibitor cocktail (Sigma-Aldrich), 1% v/v phosphatase inhibitor cocktail 2 (Sigma-Aldrich), 1% v/v phosphatase inhibitor cocktail 3 (Sigma-Aldrich), and 1 mM PMSF (Sigma-Aldrich). Protein lysates were centrifuged at 12,000 rpm at 4°C for 15 minutes to remove cell debris. Total protein concentration was determined using the Pierce BCA protein assay kit (Thermo Scientific). Following SDS-PAGE, proteins were transferred to PVDF membranes. Membranes were blocked with Tris-buffered saline containing 0.1% Tween-20 (TBST) and 5% milk for 60 minutes at room temperature. The following antibodies were diluted in TBST containing 0.5% milk, and incubated with membrane overnight at 4°C: calpastatin-pS633, calpastatin-pS351 (custom ordered from Genscript), SMC1A, SMC1A-pS957, beta-actin, tubulin, calpastatin, calpain-1, calpain-S1, myc-tag, ATM-pS1982, ATM, Chk2, and Chk2-pT68 (Cell Signaling Technology). Mouse or rabbit IgG-HRP conjugate secondary antibodies (Cell Signaling Technology) and an enzyme-linked chemiluminescence kit (Pierce) were used to detect proteins.

## SUPPLEMENTARY MATERIALS FIGURES AND TABLES



## Abbreviations

GSC: glioblastoma stem cell
NHA: normal human astrocyte
CAST: calpastatin
CK2: casein kinase 2
MS: mass spectrometry
