# Supplementary material for: Calpastatin phosphorylation regulates radiation-induced calpain activity in glioblastoma
Source: Oncotarget. 2018 Feb 19;9(18):14597–607. doi: 10.18632/oncotarget.24523 (PMC5865692; doi:10.18632/oncotarget.24523)
Supplement: Supplementary file 1 [file oncotarget-09-14597-s001.pdf]

Calpastatin phosphorylation regulates radiation-induced calpain activity in glioblastoma

SUPPLEMENTARY MATERIALS

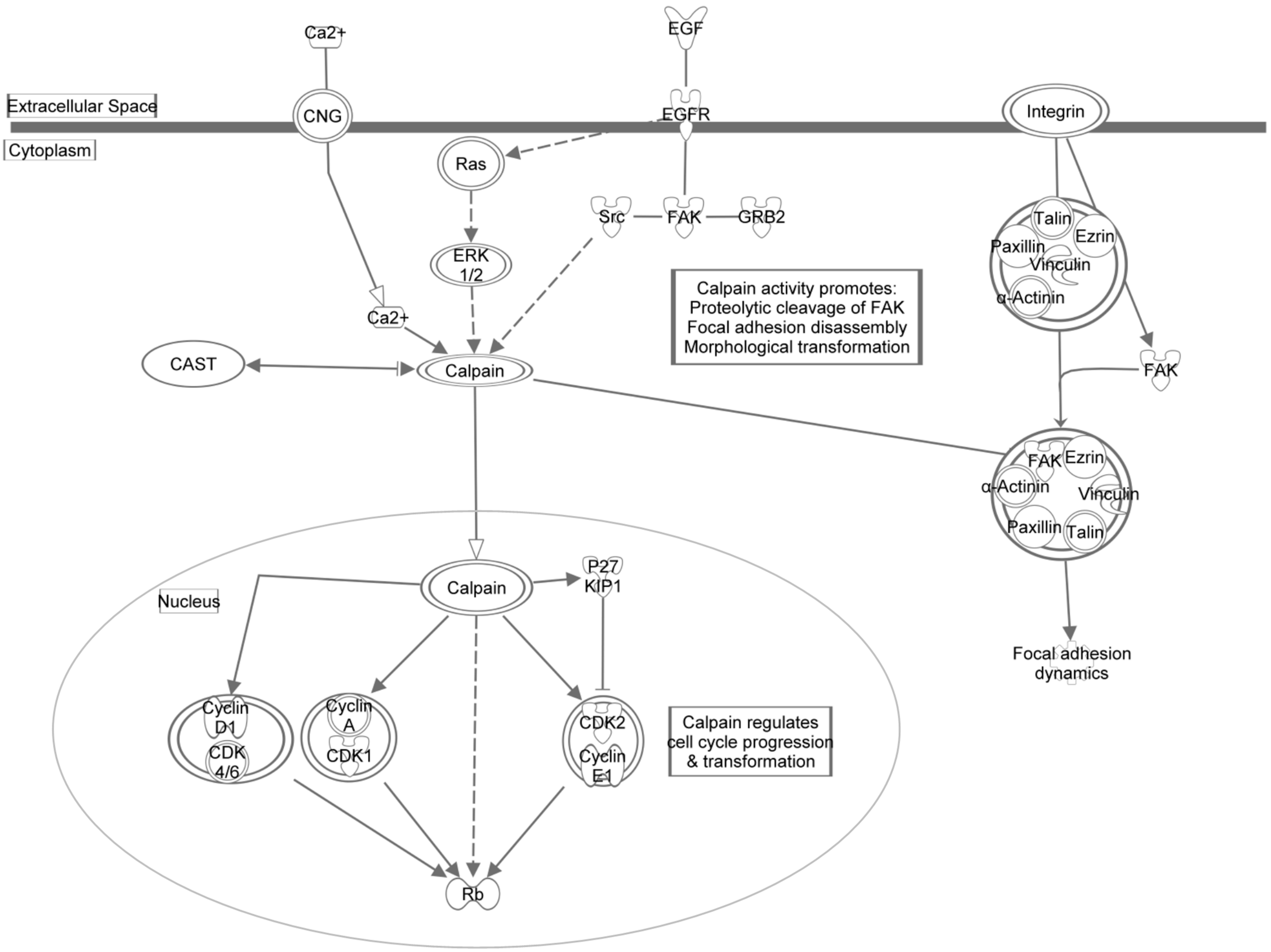

Supplementary Figure 1: Calpain signaling pathway generated from Ingenuity Pathway Analysis depicting select targets of calpain. CAST = Calpastatin.

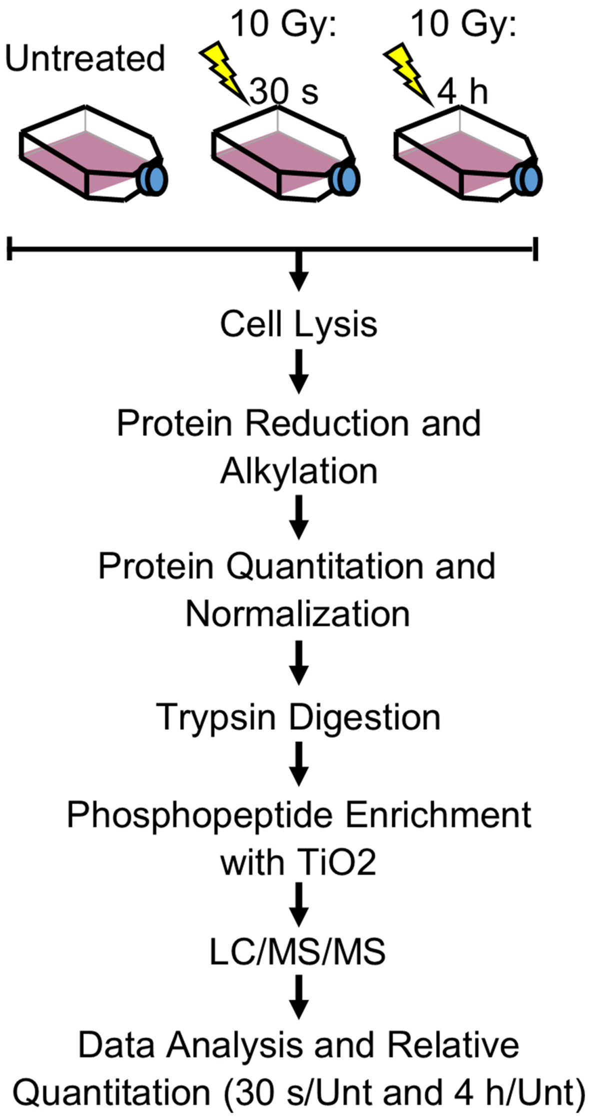

Supplementary Figure 2: Experimental scheme of the radiation treatments and sample preparation for phospho-proteomic profiling.

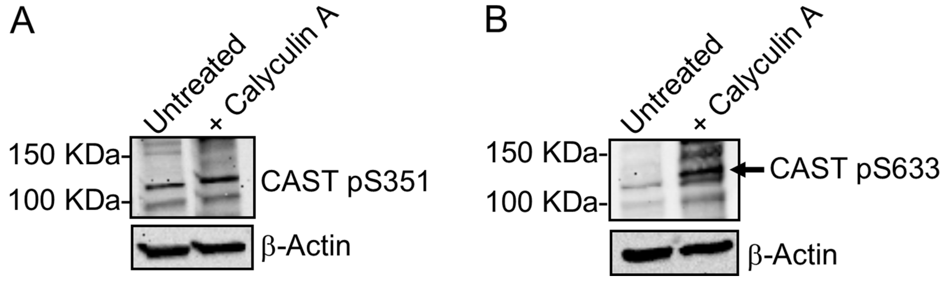

Supplementary Figure 3: Western blot validation of phospho-specific calpastatin antibodies. U87 cells were treated with the phosphatase inhibitor calyculin A to enrich for phosphorylated calpastatin. (**A**) No band specific for phosphorylated calpastatin was detected using calpastatin-pS351 antibody. (**B**) The calpastatin-pS633 antibody shows a distinct band at 120 KDa in cells treated with calyculin A. CAST = calpastatin.

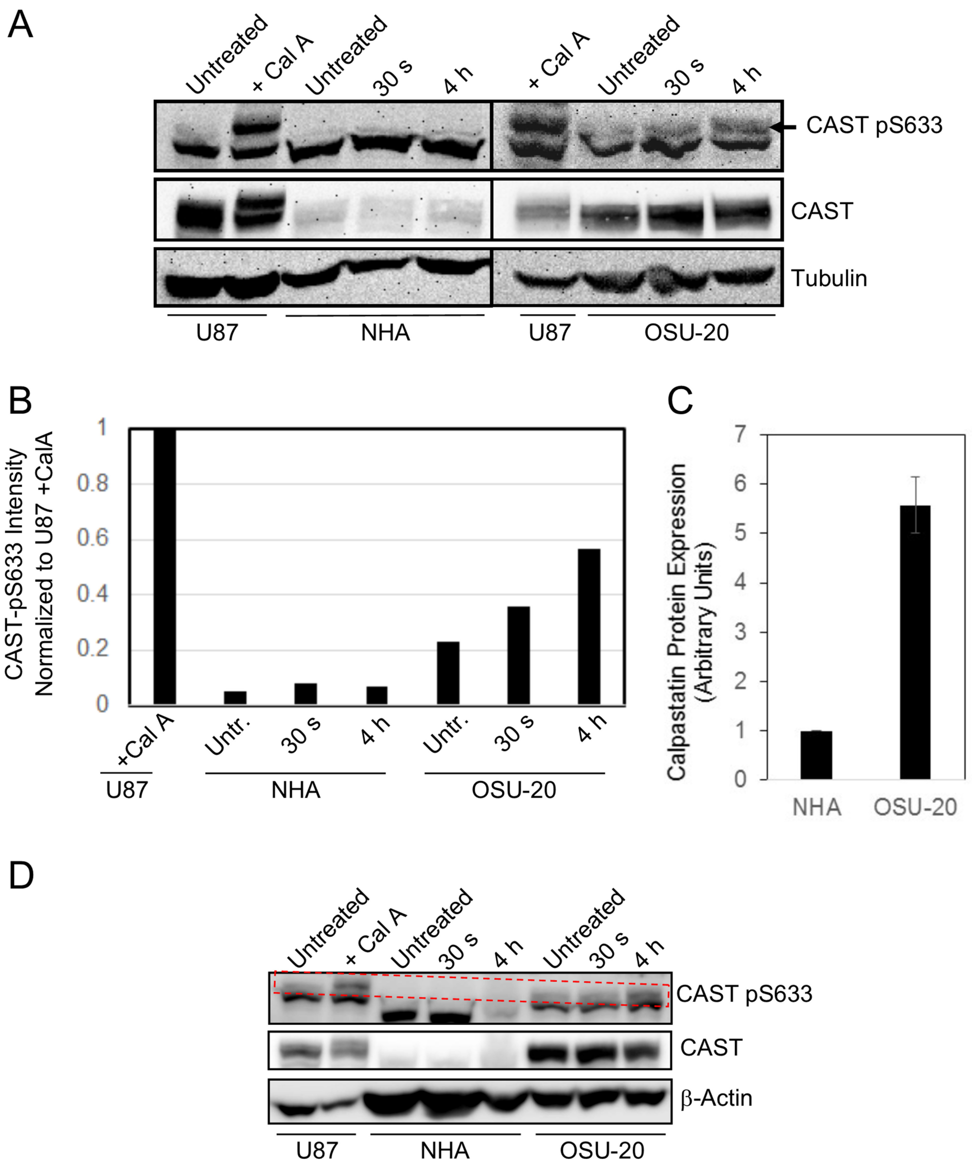

Supplementary Figure 4: Radiation-induced phosphorylation of calpastatin in GSCs. (**A**) The western blot shown in figure 2C was repeated with biological replicates to validate the result. (**B**) Quantitation of phospho-calpastatin from the western blot in (A). Calpastatin-pS633 levels in NHA and OSU-20 were normalized to Calpastatin-pS633 levels in the U87 +CalA positive control on each blot, since the two cell lines were analyzed on different western blots. (**C**) Quantitation of total calpastatin protein levels in untreated NHA and OSU-20 cells from the blot in Figure 2C. (**D**) Western blot in which twice as much protein lysate was loaded for NHA samples relative to OSU-20. The red box is used to distinguish the phospho-calpastatin band at 120 KDa from non-specific bands. CAST = calpastatin.

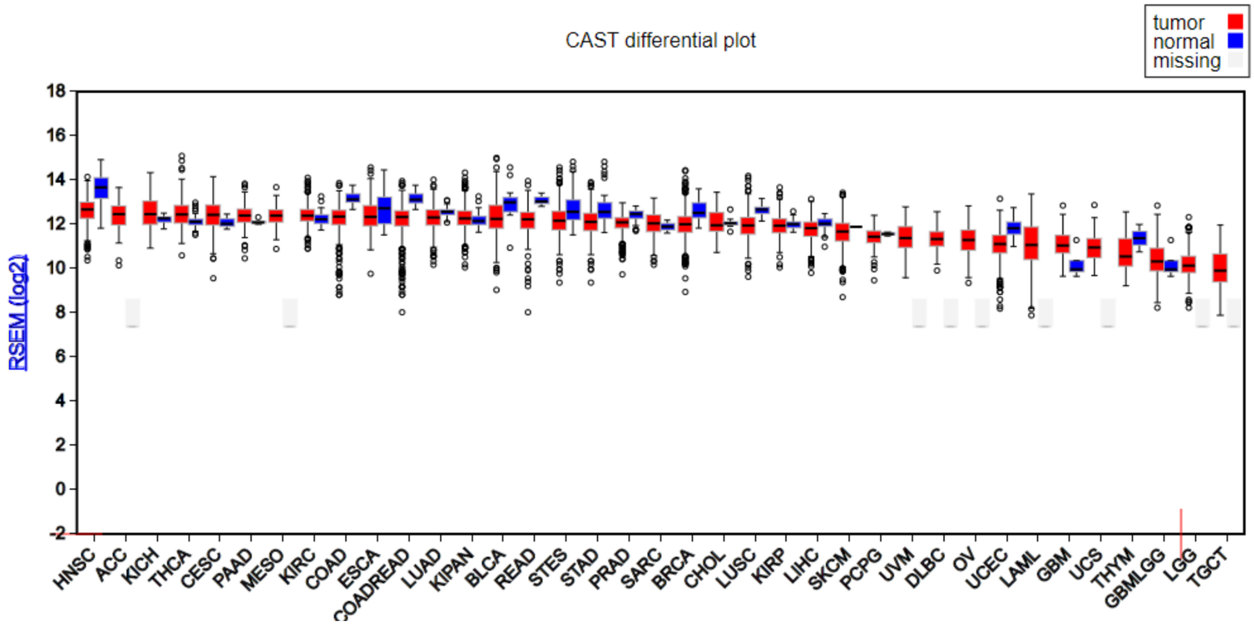

Supplementary Figure 5: CAST (calpastatin) gene expression across cancers in tumor versus normal tissue. Generated from TCGA data with the expression profile tool at firebrowse.org.

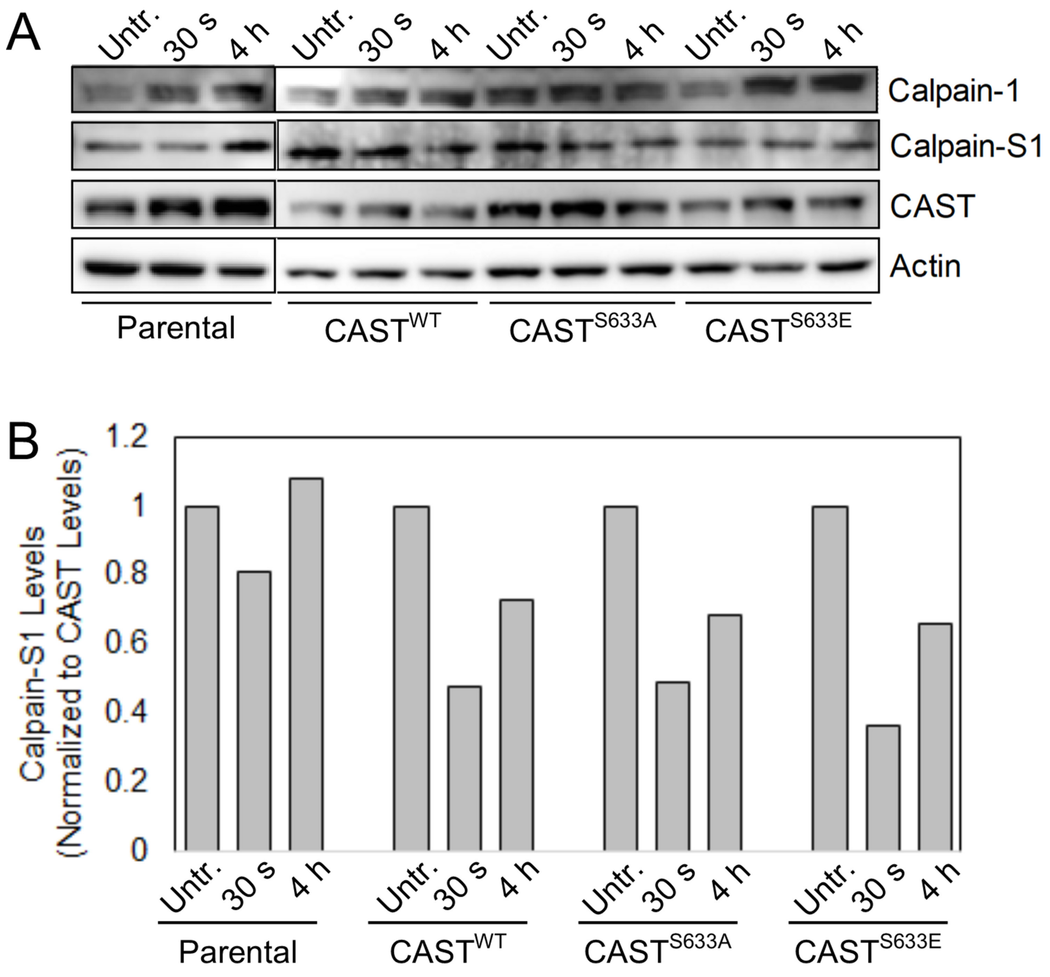

Supplementary Figure 6: Mutation of calpastatin serine 633 affects calpain-1 levels but not calpain-S1 levels following radiation treatment. (**A**) The western blot in figure 3 was repeated with biological replicates to validate the results, and to assess levels of the regulatory subunit calpain-S1. Calpain-1, calpain-S1, and calpastatin levels were assessed in LN18 parental or stable cell lines expressing calpastatin^{WT}, calpastatin^{S655A} (non-phosphorylatable mutant), or calpastatin^{S655E} (phospho-mimetic mutant). (**B**) Quantitation of calpains-S1 levels from the western blot in (A), normalized to total calpastatin levels. CAST = calpastatin.

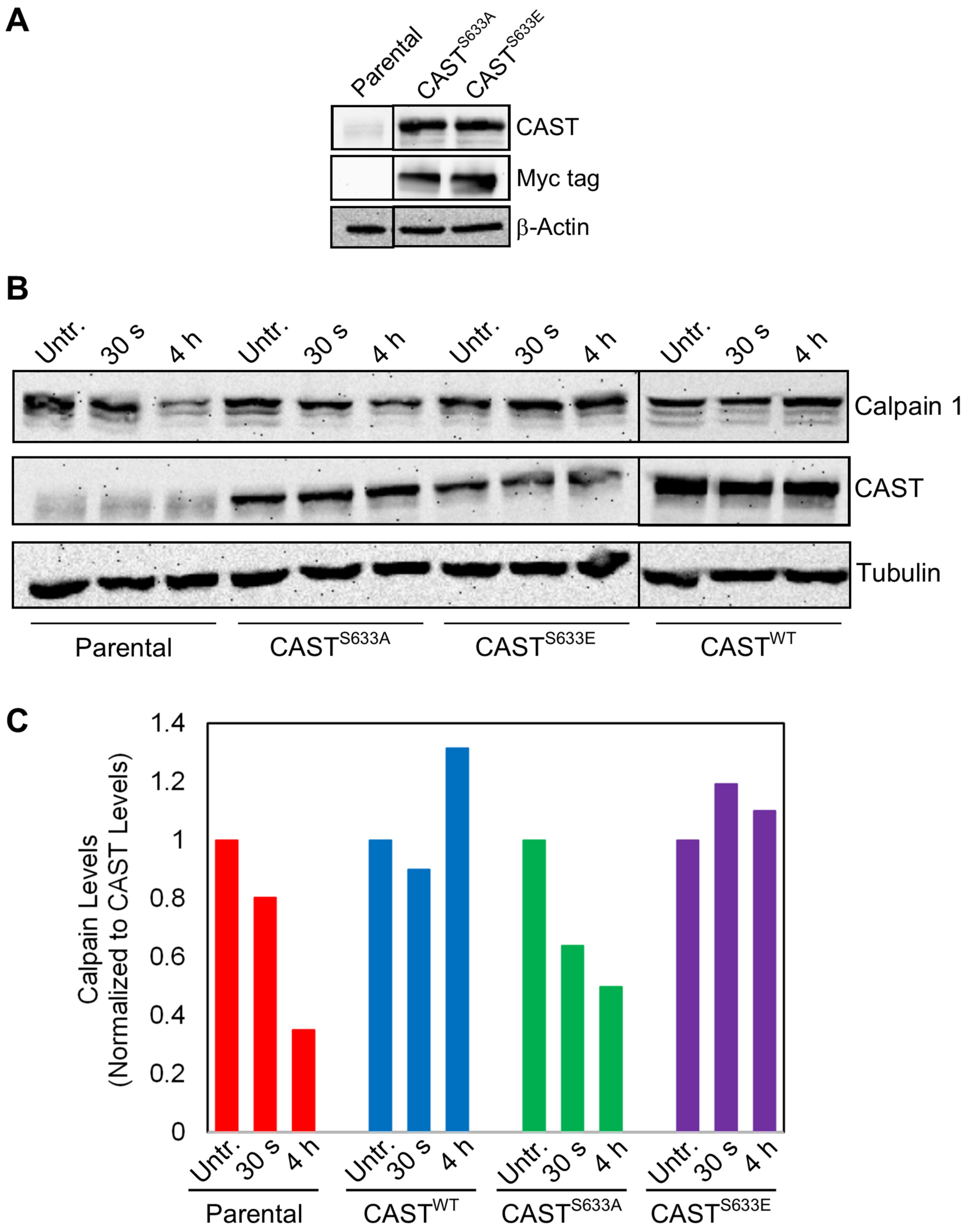

Supplementary Figure 7: Functional analysis of calpastatin phosphorylation by site-directed mutagenesis of calpastatin in U87 cells. (**A**) Stable cell lines expressing calpastatin^{S655A} (non-phosphorylatable mutant), or calpastatin^{S655E} (phospho-mimetic mutant) were generated in U87 cells. (**B**) Western blot of calpain-1 levels following radiation treatment in each of the calpastatin stable cell lines and parental LN18. (**C**) Quantitation of calpain-1 levels from the western blot from (B), normalized to total calpastatin. CAST = calpastatin.

Supplementary Table 1: References reporting radiation-induced phosphorylation of MDC1, TP53BP1, and TRIM28 at specific residues

Protein	Phospho-peptide	Phospho-site	Reference for radiation-induced phosphorylation of phospho-site
MDC1	QDG[S]QEAPEAPLSSELEPFHPKPK	S1086	Stokes et al, 2007
TP53BP1	SGTAETEPVEQDS[S]QPSLPLVR	S831	Jowsey et al, 2007
TRIM28	[S]GEGEVSGLMR	S473	Blasius et al, 2011

Supplementary Table 2: Validation of phospho-proteomic profiling with proteins known to be phosphorylated post-radiation

30 s Time-point	MDC1	TP53BP1	TRIM28
OSU-2	5.8	2.5*	0.9
OSU-11	16.0*	2.7*	1.2
OSU-20	7.2*	3.2*	1.1
U87	2.4	2.9*	0.7
4 h Time-point	MDC1	TP53BP1	TRIM28
OSU-2	5.9	1.8*	2.0*
OSU-11	6.9*	2.1*	4.9*
OSU-20	6.3*	1.9	2.4*
U87	2.1	2.6*	1.7*

Radiation-induced fold changes in phosphorylation calculated from the MS data are shown for each of the phospho-peptides in from Supplementary Table 1 across four cell lines at both time-points. The asterisk indicates a p-value <0.05 calculated from biological triplicates.

Supplementary Table 3: Post-radiation fold changes in calpastatin phosphorylation at serines 351 and 633 in OSU-53 GSCs

Calpastatin Phospho-peptide	Phosphosite	Post-RT Time-point	OSU-53
KPADDQDPIDAL[S]GDLDSCPSTTETSQNTAK	S633	30 s	1.3
KPADDQDPIDAL[S]GDLDSCPSTTETSQNTAK	S633	4 h	3.8*
SESELIDEL[S]EDFDR	S351	30 s	5.7
SESELIDEL[S]EDFDR	S351	4 h	10.3*

Fold changes were calculated for each time-point (30 s/Untreated and 4 h/Untreated) from triplicate intensity values from mass spectrometry data.
